# Yes, Sex and Gender Have Separate Identities!

**DOI:** 10.1007/s10508-025-03164-9

**Published:** 2025-08-11

**Authors:** James G. Pfaus, Ellen Zakreski, Gonzalo R. Quintana

**Affiliations:** 1 Department of Psychology and Life Sciences, Faculty of Humanities, Charles University, 18200 Prague, Czech Republic; 2 Center for Sexual Health and Interventions, Czech National Institute of Mental Health, Klecany, Czech Republic; 3 Departamento de Psicología, Facultad de Ciencias Sociales, Universidad de Chile, Santiago, Chile

Joe Pyne: “So, Frank, you have long hair. Does that make you a woman?”

Frank Zappa: “So, Joe, you have a wooden leg. Does that make you a table?”

--Frank Zappa interviewed on the Joe Pyne Show, 1966 (Zappa & Occhiogrosso, 1989).

We have argued previously that the conflation of biological sex with socially (and personally) constructed gender has done a world of damage by pitting biology against culture and nature against nurture (with a biological and experience-dependent brain trying to make sense of the two). This has resulted in conflicting social movements that deny science while trying to dominate one another (Quintana & Pfaus, 2024). As a reaction to what the current conservative administration in the USA describes as a “liberal” social justice movement that was undermining the moral purity of the nation, the government dialed back nearly 60 years of true reform and legal protection for sex, gender, and other minorities. A similar imposition of conservative moral values around sex and gender has occurred in some European, African, and Asian nations with similar regime ideologies. Recently in the USA, ongoing funding for the scientific study of sex and gender has been abruptly and egregiously halted because such research “does not improve the quality of life for the American taxpayer.” Grant applications with newly forbidden terms like “sex,” “gender,” “woman,” and “female” (among a huge list) are being refused outright. This leaves sexual science in a profound quandary.

**Fig. 1:**
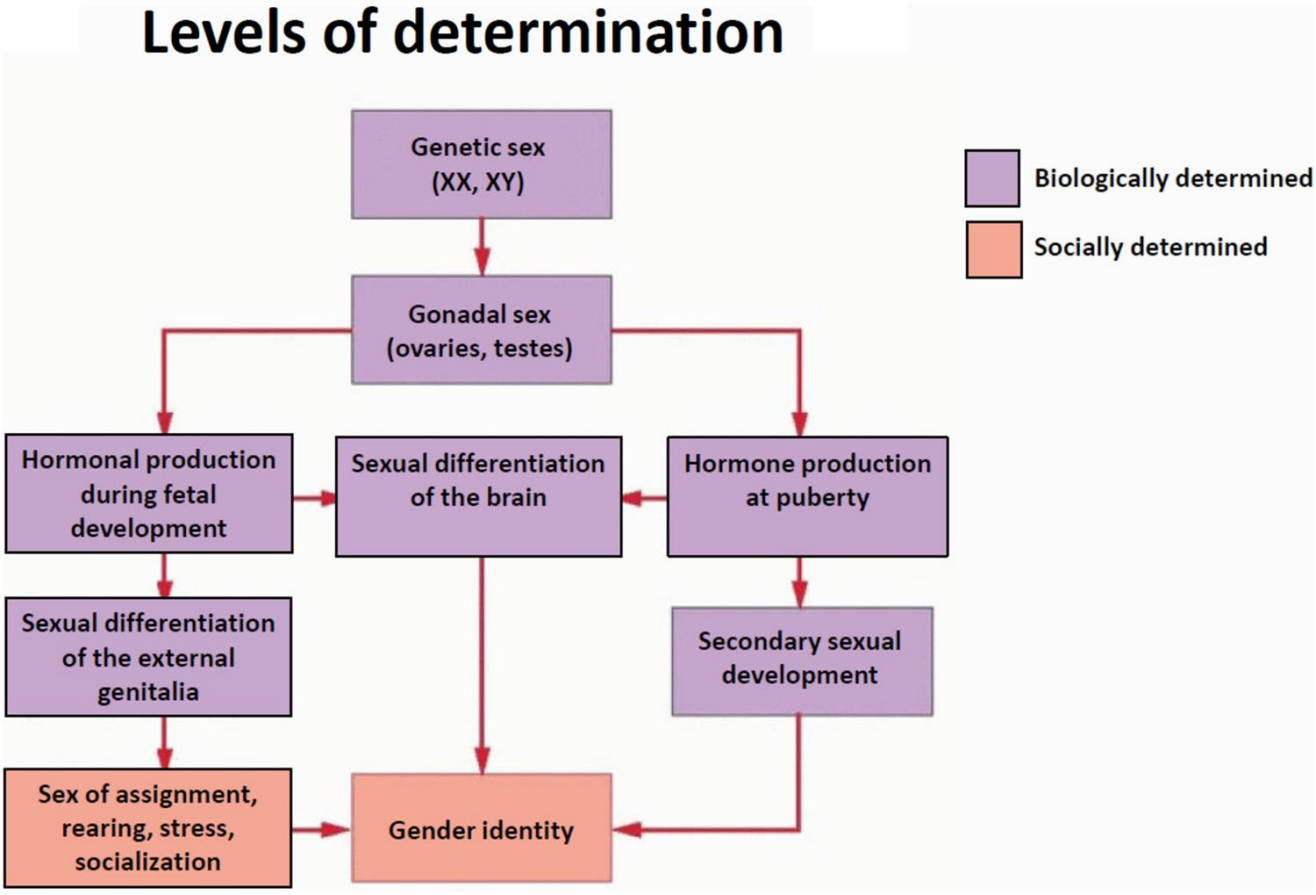
Levels of determination of biological sex and socially constructed gender. Adapted with permission from OBGYN Key: Normal and Abnormal sexual development. https://obgynkey.com/normal-and-abnormal-sexual-development/

On the one hand, we understand the desire of human beings to derive a singular, meta-cognitive appraisal of who they are at any one point in time that binds all the different biological, social, and even political parts together into a congruent “whole.” Thus, we have concepts like “gender/sex” and “sexual configurations” (van Anders, 2015) that reflect this confluence, much like a particular hue of ink after it is dissolved in water. But on the other hand, conflating the parts gives rise to the false notion that they are both interchangeable, if not inseparable. Ink and water are still composed of separate molecules. The parts may have additive or multiplicative relationships in different people, with some parts changing while others stay stable in time. This makes it difficult, if not impossible, to scale them together as a singularity in any objective way. Nevertheless, it is simple for people to assume that genetic or chromosomal sex is normally congruent with gonadal structure, steroid hormone output, gamete production, secondary sex characteristics related to reproductive status, and sex differences in brain structure and function (e.g., McCarthy et al., 2012) that resemble a “mosaic” of more-or-less female and male phenotypes (e.g., Joel 2021). The expression of gender relies on these factors along with family rearing, stress, and socialization processes (Fig. 1).

Certainly, from a biologically deterministic perspective, sex serves reproduction, and sexual reproduction requires a merging of DNA carried by chromosomes within parental gametes of egg and sperm. Therefore, with few exceptions, more than 99% of humans, like other mammals, are born either female or male, with ovaries or testes, respectively, that produce sex-specific gametes. The gonads also produce sex-specific patterns of steroid hormone release in utero that sculpts the gonads, brain, and body into male or female phenotypes, and in adulthood that primes the brain for sexual function, i.e., desire, arousal, sexual behavior, and pleasure. However, from a sociological perspective, the binary conception of men and women as somehow biologically fixed extensions of female or male genetic or chromosomal sex has been challenged. In particular, the socially constructed concept of “gender” has replaced what used to be referred to as “sex roles” (Maccoby & Jacklin, 1974) with a fluid spectrum that includes culturally defined expressions of masculinity and femininity, as well as both together in varying degrees, trans, or neither (see Haig 2000, 2004).

We have argued that sex is largely, if not exclusively, defined in terms of DNA and chromosomal structure, but that it is also influenced by hormonal makeup, and typically determined by the anatomy and physiology of external genital structures (e.g., clitoris, vagina, penis) that are sexually differentiated in utero and observed on ultrasound and/or revealed at birth and “assigned” on birth records (Quintana & Pfaus, 2024). Approximately 1% of individuals exist with intersex conditions, or rare disorders of sexual development (DSDs) that can appear at birth as incomplete genital development. These can be due to androgen insensitivity syndrome (AIS), an androgen receptor mutation in genetic males that demasculinizes them, congenital adrenal hyperplasia (CAH) in genetic females which androgenizes and defeminizes them, along with mixed genital dysgenesis that results in incomplete genital development in both. Some of these conditions (e.g., AIS) are not revealed until puberty or when people attempt to reproduce. Indeed, some intersex individuals can make viable gametes while others cannot. Although some have argued that intersex individuals constitute a “third sex” (third or more, see e.g., Fausto-Sterling 2019), there are no intersex/DSD specific genes or chromosomes in these individuals that support a third type of gamete. Importantly, neither sex nor DSDs predict gender congruence or incongruence.

Gender has to do with people’s feelings and expressions of femininity and masculinity (e.g., Money 1987). In our previous paper (Quintana & Pfaus, 2024), we argued that orthodox Judeo-Christian-Islamic religious traditions, along with European cultural perceptions, have always conflated gender with sex: two sexes meant two genders and their appropriate “feminine” and “masculine” traits that included goals, abilities, appearance, clothing, mannerisms, behaviors (especially sexual behavior and sexual orientation), social status, and self-identification. We argue here that this conflation forms the foundation of the current socio-political quagmire. Last century, girls who displayed more “masculine” traits (everything from wearing pants to having short hair and doing traditionally masculine things like climbing trees or flying airplanes) were considered abnormal and called “tomboys” among other names. They were likely to reject the social norm of what it meant to be a “feminine woman” and thereby rejected the social controls imposed by religious and cultural institutions, especially regarding sexual and reproductive traditions like marriage, maternity, and motherhood. Some were more likely to engage in homosexual behavior and adopt a homosexual orientation. Some went further and incorporated masculine traits completely, living as “sexually-inverted” (trans) men. Research in the mid-twentieth century established that girls with CAH were far more likely to display culturally masculine traits, and it was assumed that this was because they had been androgenized in utero (Kung et al., 2024; Money & Ehrhardt, 1972). Analogous behavioral results were found in female monkeys who had been androgenized during the critical perinatal organizational period (Goy, 1970), displaying male-like traits of rough-and-tumble play and threat postures.

Likewise, boys who displayed more “feminine” traits were considered abnormal for identical reasons that spanned the rejection of cultural masculinity, potential display of a homosexual orientation, and a “sexual inversion” in which they adopted feminine traits and perhaps lived as (trans) women. However, other cultures (e.g., the indigenous peoples of North America) did not conflate sex and gender. Many indigenous cultures (Iroquois, Hunkpapa Lakota, Sac, and Fox nations) recognized at least three genders (female, male, both), while some, like the Cree, recognized at least six: “Iskwêhkân” (a woman who acts/lives as a woman), “Napêhkân” (a man who acts/lives as a man), “Iskwêw ka napêwayat” (a woman who dresses as a man), “Napêw iskwêwisêhot” (a man who dresses as a woman), “Înahpîkasoht” (a woman dressed/living/accepted as a man), and “Ayahkwêw” (a man dressed/living/accepted as a woman (2-Spirited People, 2021). If we add Two Spirited to this, we get seven. But gender expression is a fluid concept. The long hair of the Beatles, Frank Zappa, and others during the 1960s was considered by many conservatives to be a rejection of traditional masculinity, an expression of femininity, or both. It was “dangerous” in its freedom, something so cogently expressed in the musical *Hair* (MacDermot, 1966). Yet, a decade later, long hair worn by men had been normalized everywhere in the West. And as a foreshadow to our current situation, never forget actor James Dean playing teenager Jim Stark in the film *Rebel Without A Cause* (1955), seeking desperately to be a “real man,” confronting his passive and beleaguered father (actor Jim Backus) because he wore an apron in the kitchen.

In addition to the disruption of gender stereotypes by cis-gendered individuals during this era, trans-persons had already started to receive psychiatric and medical care. The first male-to-female sex-change operations in Germany in the 1920s involved surgical removal of the gonads (Rivera, 2024). This was followed by the addition of female-like or male-like sex hormone replacement in the 1950s, with ongoing refinement of plastic surgery to craft genitals of the opposite sex. This has allowed gender dysphoric individuals in the current day to dress, act, and look like the opposite sex to varying degrees, thus reducing or eliminating their dysphoria and contributing to a better quality of life. Despite this, we are no closer to understanding how cis- or trans-gender identities develop biologically. Some studies examining the brains of trans-females have reported female-like morphology (e.g., size of the central subdivision of the bed nucleus of the stria terminalis; Zhou et al., 1995) or function (e.g., kisspeptin expression in the infundibular (arcuate) nucleus of the hypothalamus; Taziaux et al., 2016). Although intriguing, these studies are typically conducted on individuals who already transitioned and had been on female hormone replacement. Thus, it is not known whether the differences preceded the hormone replacement or developed from it.

Since the early 2000s, gender identity politics have spawned many special interest groups with idiosyncratic definitions of gender, sex, and sexual orientation. For many, gender and sex are completely conflated, leading to a rejection of a biologically determined female-male binary in favor of a socially constructed rainbow of conflated, “nonbinary” gender/sex variations. Although these groups formed with a common purpose of social justice, riding perhaps on the coat-tails of the feminist and gay/lesbian civil rights movements and nurtured by social constructionists, their interactions have devolved around whose identity politics are correct. For example, in the past decade, we have seen the rise of rapid onset gender dysphoria in a subpopulation of largely adolescent females who did not experience gender dysphoria in childhood, but nevertheless suddenly want to be trans-males (e.g., Leonhardt et al., 2025, Littman, 2018). This has pitted some lesbian feminists against certain trans-groups that accuse them of being “trans exclusionary” because they do not believe that all lesbians are actually closeted trans-males. And in turn, these cultural flame wars have led to a backlash among conservatives who also conflate sex and gender, but pine for the “good old days” when father knew best, when actor John Wayne was in the saddle; when “men were men” (and women were not) and pronouns were simple.

Donald Trump’s Executive Order on 20 January 2025, stated: “It is the policy of the United States to recognize two sexes, male and female.” And later, “‘Sex’ is not a synonym for, and does not include, the concept of ‘gender identity.’” Although we disagree vehemently with virtually all of Trump’s agenda, we acknowledge the old saying that “Even a stopped clock is right twice a day” (von Ebner-Eschenbach, 1880). If “two sexes” refers to the presence of functional ovaries or testes at birth that can make sexually differentiated gametes for reproduction, then we agree, even if those gonads are removed in adulthood. And if “gender identity” refers to a socially and personally constructed view of sex and gender roles, dress, actions, along with altered body morphology, etc., then we also agree that sex is not synonymous with it. But that goes both ways: Gender is not synonymous with sex. There are not “two genders” and one’s sex should not be taken de facto as one’s gender. The two are separate parts of a person, even if they are consistent in most human beings. Trans-persons exist, and they do so in varying degrees of diverse expression, from absolute binary to subtle modes of nonbinary and genderqueer to Two Spirited. They always have.

The fact that sex and gender are *separate* parts of a person should not come as an affront to anyone. Breastfeeding is something that natal females do after giving birth, even as trans-males, unless their breasts have been removed. Calling it “chestfeeding” in order to placate someone’s refutation of being female is absurd and unnecessary. The short-term gain in doing so will always be followed by the long-term pain of science denial. Your sex is female, your gender is masculine. Simple. An extra space for gender could even be put on government IDs. However, the distinction between gender and sex should never be weaponized against trans-persons and their access to gender-affirming care. This includes access to safe surgical body modification (e.g., vaginoplasty, clitoroplasty, phalloplasty, breast enhancement or removal, etc.) that maintains the integrity of genitopelvic nerves, along with access to different types of hormone replacement that suit the needs of each individual. It also requires the continued development of therapeutic interventions by trained psychologists and sexologists to help guide trans-persons to a healthy endpoint that includes sexual pleasure and satisfaction. It requires therapists to distinguish transitioners from those at risk for detransition, and to help them, their families, and loved ones adjust to reality. It requires all of us to openly and respectfully consider empirical evidence of things that some of us might not like, such as autogynephilia. All of this requires basic and clinical research on sex and gender to continue to move forward, objectively, with proper peer review and funding, and without political interference guided by ideology.

How many sexes are there? In humans, two that are functional from a reproductive standpoint. How many genders are there? At least six or seven, as the Cree elders suggested, but probably with as many subtle differences in definition and co-morbidity as there are people on Earth.
